# Making a C-DIFFerence: Implementation of a prevention collaborative to reduce hospital-onset *Clostridioides difficile* infection rates

**DOI:** 10.1017/ash.2022.54

**Published:** 2022-05-26

**Authors:** Katelyn A. White, Laura E.A. Barnes, Rachel L. Snyder, Lucy V. Fike, David T. Kuhar, Ronda L. Cochran

**Affiliations:** Division of Healthcare Quality Promotion, Centers for Disease Control and Prevention, Atlanta, Georgia

## Abstract

**Objective:**

To assist hospitals in reducing *Clostridioides difficile* infections (CDI), the Centers for Disease Control and Prevention (CDC) implemented a collaborative using the CDC CDI prevention strategies and the Targeted Assessment for Prevention (TAP) Strategy as foundational frameworks.

**Setting:**

Acute-care hospitals.

**Methods:**

We invited 400 hospitals with the highest cumulative attributable differences (CADs) to the 12-month collaborative, with monthly webinars, coaching calls, and deployment of the CDC CDI TAP facility assessments. Infection prevention barriers, gaps identified, and interventions implemented were qualitatively coded by categorizing them to respective CDI prevention strategies. Standardized infection ratios (SIRs) were reviewed to measure outcomes.

**Results:**

Overall, 76 hospitals participated, most often reporting CDI testing as their greatest barrier to achieving reduction (61%). In total, 5,673 TAP assessments were collected across 46 (61%) hospitals. Most hospitals (98%) identified at least 1 gap related to testing and at least 1 gap related to infrastructure to support prevention. Among 14 follow-up hospitals, 64% implemented interventions related to infrastructure to support prevention (eg, establishing champions, reviewing individual CDIs) and 86% implemented testing interventions (eg, 2-step testing, testing algorithms). The SIR decrease between the pre-collaborative and post-collaborative periods was significant among participants (16.7%; *P* < .001) but less than that among nonparticipants (25.1%; *P* < .001).

**Conclusions:**

This article describes gaps identified and interventions implemented during a comprehensive CDI prevention collaborative in targeted hospitals, highlighting potential future areas of focus for CDI prevention efforts as well as reported challenges and barriers to prevention of one of the most common healthcare-associated infections affecting hospitals and patients nationwide.

*Clostridioides difficile* infection (CDI) remains one of the most common healthcare-associated infections (HAIs). Acute-care hospitals (ACHs) in the United States saw an 18% decrease in hospital-onset CDI between 2018 and 2019, as well as a steady decline in the standardized infection ratio (SIR) from 0.63 to 0.55 during 2019.^1^ However, these decreases may be leveling off; national CDI SIRs remained stable at 0.52 throughout 2020.^1,2^

To guide prevention efforts, the Centers for Disease Control and Prevention (CDC) developed the CDI prevention strategies, which provide basic interventions recommended for the prevention of CDI. These include 5 strategies: (1) isolate and initiate Contact Precautions for suspected or confirmed CDI, (2) confirm CDI in patients, (3) perform environmental cleaning to prevent CDI, (4) develop infrastructure to support CDI prevention, and (5) engage the facility antibiotic stewardship program.^3^

These CDI prevention strategies are integrated into the CDC Targeted Assessment for Prevention (TAP) Strategy, a quality improvement framework that uses data for action to guide HAI prevention. The TAP Strategy consists of 3 components: (1) targeting facilities and/or units with the greatest opportunity for improvement, (2) assessing targeted locations to identify infection prevention gaps, and (3) addressing gaps by implementing tailored interventions.^4^

Using the CDI prevention strategies and the TAP Strategy as foundational frameworks, the CDC implemented the Let’s Make a C-DIFFerence CDI Prevention Collaborative from October 2019 through September 2020. This national collaborative was conducted with support from the American Hospital Association (AHA). ACHs from across the country enrolled with the aim of improving infection prevention practices and reducing CDI rates. This article describes the collaborative experience and summarizes infection prevention barriers, gaps identified, interventions implemented, and SIR outcomes.^5^

## Methods

### Collaborative recruitment

ACHs were identified for recruitment using CDI laboratory-identified data reported to the National Healthcare Safety Network (NHSN) from October 2017 to September 2018 using the cumulative attributable difference (CAD). The CAD is the primary metric of NHSN TAP Reports and can be used to rank facilities based on their number of excess infections above an SIR goal.^6^ Among ACHs with SIRs >0.90 (75th percentile of hospitals by SIR), the 400 hospitals (or 10.9% of 3,675 reporting) with the highest CADs were identified for recruitment; 18 were excluded due to active involvement in other CDC CDI prevention projects. During June and July 2019, 382 ACHs were contacted for recruitment. Communications were directed to NHSN administrators, chief medical officers, and quality contacts.

### Collaborative experience

The collaborative commenced in October 2019 with a kick-off meeting in Chicago, Illinois. Collaborative participants at each hospital primarily consisted of infection preventionists. Monthly webinars focusing on CDI prevention strategies presented by national subject-matter experts were provided live. Dedicated office hours were hosted monthly, and one-on-one coaching calls were available for participants to communicate with CDC subject-matter experts for guidance on TAP implementation and CDI prevention efforts. A collaborative e-mail ListSERV was also utilized to allow sharing among participants. All webinar recordings and materials related to the collaborative were available for participants to access any time via SharePoint. A final virtual meeting was conducted in September 2020 via Adobe Connect.

### TAP Strategy implementation

CDI TAP facility assessments were deployed among frontline staff to garner awareness and perceptions of prevention policies and practices to identify opportunities for improvement. Assessments were available via paper, SurveyMonkey, REDCap, and/or fillable PDF. Separate assessments specific for environmental services (EVS) staff were available in English and Spanish, and optional laboratory assessments were available for completion by the laboratory manager.

### Outcomes

The CDC provided Feedback Reports summarizing assessment results and guided hospitals in interpreting the data to identify gaps. Hospitals utilized Gap Prioritization Worksheets to prioritize gaps and communicate previous prevention efforts back to the CDC.^4^ With this information, the CDC created customized hospital profiles for each participating hospital, summarizing their reported prevention strengths and barriers, previously implemented interventions, and assessment results, and providing tailored feedback, recommended strategies, and example tools to guide interventions.

After the collaborative in August 2021, calls were conducted with 14 of 15 hospitals that completed the TAP Strategy (ie, collected assessments, reviewed results, submitted priority gaps) and were most consistently engaged throughout the collaborative.

Demographic and CDI laboratory-identified data from NHSN were reviewed for participating hospitals and hospitals that met the recruitment criteria but did not participate (ie, nonparticipants). SIRs were calculated by quarter, with 4 quarters per period: pre-recruitment (October 2017–September 2018), pre-collaborative (October 2018–September 2019), during the collaborative (October 2019–September 2020), and post-collaborative (October 2020–September 2021). Mid-*P* exact tests were used to compare SIRs between the pre-collaborative and post-collaborative periods. The χ^2^ test was used to examine the relationship between participants and nonparticipants and their improvement from a positive CAD in the pre-collaborative period to a CAD of zero or less in the post-collaborative period. The *χ* ^2^ test, Fisher exact test, and Wilcoxon 2-sample test were used as appropriate to compare hospital characteristics. *P* values < .05 were considered statistically significant.

### Data sources

Hospital demographics, contact information, and CDI data were obtained through the NHSN. Other hospital-specific information obtained through various collaborative activities was qualitatively coded by categorizing each response to the respective CDI prevention strategy domains. Data were analyzed using Microsoft Excel (Microsoft, Redmond, WA), NVivo (QSR International, Melbourne, Australia), and SAS version 9.4 software (SAS Institute, Cary, NC).

## Results

### Collaborative recruitment

In total, 76 hospitals were successfully enrolled, 30 (39.5%) of which were in the top 100 based on their CADs, representing hospitals with the highest burden of excess infections. Participating hospitals spanned 30 states and most (92%) were general ACHs; additional hospital characteristics are described in Table 1.

**Table 1.** Hospital Characteristics Among Collaborative Participant Hospitals (n = 76) and Hospitals That Met the Recruitment Criteria but Did Not Participate (Nonparticipants, n = 306).

Characteristic	Participants (N = 76), No. (%)	Nonparticipants (N = 306), No. (%)	*P* Value
Medical school affiliation	66 (86.8)	249 (81.4)	.262
**Hospital size**			
≤200 beds	20 (26.3)	85 (27.8)	.551
201–500 beds	40 (52.6)	175 (57.2)	
501–1,000 beds	15 (19.7)	43 (14.1)	
>1,000 beds	1 (1.3)	3 (1.0)	
On-site laboratory	65 (85.5)	233 (76.1)	.077
CDI Admissions (2018 Quarter 1), median (IQR)	3,660 (2,254–6,578)	3,592 (2,370–4,998)	.392
**CDI test type** ^a^ **(2018 Quarter 1)**			
EIA	15 (19.7)	54 (17.6)	.892
NAAT	60 (78.9)	248 (81.0)	
Other	1 (1.3)	4 (1.3)	

Note. CDI, *Clostridioides difficile* infection; IQR, interquartile range; EIA, enzyme immunoassay; NAAT, nucleic acid amplification test; GDH, glutamate dehydrogenase.

a CDI test type was categorized as follows: NAAT includes NAAT, GDH + NAAT, and GDH + EIA + NAAT; EIA for toxin includes EIA for toxin, GDH antigen + EIA for toxin, and NAAT + EIA; and Other includes all other CDI test types, including the selection of “Other” and associated free-text entry.

### Collaborative experience

Prior to the start of the collaborative, 45 hospitals (61%) reported that practices related to CDI testing were among their greatest barriers and/or challenges to prevention (eg, provider understanding of and adherence to appropriate testing practices, timeliness of ordering and specimen collection). Furthermore, 38 hospitals (53%) were most interested in learning about testing practices (eg, testing algorithms, optimal testing methods, education and engagement of staff) (Table 2).

**Table 2.** Qualitative Information Provided by Participants Throughout the Collaborative, Categorized to Align With the *Clostridioides difficile* (CDI) Prevention Strategies

Question	No. of Hospitals	Strategy 1: Isolate and Initiate Contact Precautions for Suspected or Confirmed CDI, %	Strategy 2: Confirm CDI in Patients, %	Strategy 3: Perform Environmental Cleaning to Prevent CDI, %	Strategy 4: Develop Infrastructure to Support CDI Prevention, %	Strategy 5: Engage the Facility Antibiotic Stewardship Program, %	Other
What is the greatest barrier or challenge to CDI prevention at your facility?^a^	74	20	61	9	14	16	28
What CDI topic areas are you most interested in learning about during this Collaborative?^a^	72	13	53	28	21	22	50
Which prevention strategy is your facility’s greatest strength?	68	46	18	18	7	12	…
Gaps prioritized by facilities after review of CDI TAP Facility Assessment results^a^	15	87	93	93	100	73	…
What CDI prevention efforts did your facility implement (or does your facility plan to implement) as a result of participation in the Collaborative?^a^	14	43	86	50	64	43	21
Share an example of how your facility has made a C-DIFFerence during this Collaborative^a^	28	18	50	14	54	11	11

Note. CDI, *Clostridioides difficile* infection; TAP, targeted assessment for prevention.

a Open-ended responses were qualitatively coded to align with the CDI prevention strategies. Hospitals may have provided >1 distinct item for select questions and hospital responses may be counted under >1 strategy.

### TAP Strategy implementation

In total, 5,673 CDI TAP facility assessments were collected across 46 hospitals (61%) during varying periods from November 2019 to August 2020, with a median of 61 assessments (IQR: 25, 187) per hospital. Most (59%) participants collected assessments from across their hospitals, while others identified specific units to target based on their CDI data and contextual factors. The most common respondents included nurses (53.2%); ancillary staff (12.3%); certified nurse assistants, patient care technicians, or patient care assistants (10.3%); and physicians, physician assistants, or nurse practitioners (6.0%). In total, 581 EVS assessments were collected by 20 hospitals (26%) with a median of 21.5 (IQR: 11, 41) assessments per hospital. Overall, 21 hospitals (28%) completed the laboratory assessment.

Questions were identified as potential gaps at the hospital level if ≥33% of respondents answered “unknown”, or if ≥50% answered “no" or "unknown”, or “never”, “rarely”, “sometimes”, or “unknown". Items for which at least 50% of the hospitals identified a gap from the CDI TAP facility assessment are presented in Table 3 and from the EVS assessment presented in Table 4.

**Table 3.** *Clostridioides difficile* Infection (CDI) Targeted Assessment for Prevention (TAP) Facility Assessment Questions for which at Least 50% of Hospitals Identified a Gap^a^

Question	% of Hospitals		
**CDI Prevention Strategy 1: Isolate and initiate Contact Precautions for suspected or confirmed CDI**	**Gap** ^b^	**Neutral**	**Strength** ^c^
Is CDI status (ie, suspected, confirmed, and recent history) communicated from other facilities upon transfer to your facility?	89	9	2
Is CDI status (ie, suspected, confirmed, and recent history) communicated to receiving facilities upon transfer from your facility?	59	24	17
Do patients with CDI remain on Contact Precautions for at least 48 hours after diarrhea ends?	54	28	17
Do patients with CDI receive daily baths/showers with soap and water?	76	13	11
Is there a process in place to ensure that patients perform hand washing after using the bathroom?	72	26	2
Is there a process in place to ensure that patients perform hand washing before eating?	83	17	0
Do families/visitors adhere to use of gowns/gloves for patients on Contact Precautions?	87	9	4
Do families/visitors adhere to hand hygiene policies?	93	7	0
**CDI Prevention Strategy 2: Confirm CDI in patients**	**Gap**	**Neutral**	**Strength**
Do ordering providers document an indication for *C. difficile* tests?	70	24	7
Do providers avoid ordering *C. difficile* tests when the patient has a known cause for diarrhea (eg, history of laxatives)?	87	11	2
Do providers avoid testing for cure of CDI?	98	0	2
**CDI Prevention Strategy 3: Perform environmental cleaning to prevent CDI**	**Gap**	**Neutral**	**Strength**
Are high-touch environmental surfaces (eg, bed rails/controls, tray table) in patient rooms cleaned daily?	57	39	4
Is it clear which items are cleaned by environmental services personnel versus other personnel (eg, nurses, nursing assistants, clerks)?	57	37	7
Is an EPA-registered product that is effective against *C. difficile* spores used for daily disinfection in the rooms of patients with CDI?	61	30	9
Is enough time provided for personnel to complete postdischarge (terminal) cleaning of patient rooms?	52	37	11
Are disinfectants used according to the instructions on the label (eg, contact time, precleaning)?	61	26	13
**CDI Prevention Strategy 4: Develop infrastructure to support CDI prevention**	**Gap**	**Neutral**	**Strength**
Does your facility have a staff person with dedicated time to coordinate CDI prevention activities?	87	9	4
Does your facility have a nurse champion for CDI prevention activities?	91	4	4
Does your facility have a physician champion for CDI prevention activities?	98	0	2
Does your facility routinely audit environmental cleaning/disinfection for personnel with this responsibility?	80	13	7
Does your facility routinely provide feedback on performance of environmental cleaning and disinfection to personnel with this responsibility?	76	17	7
Does your facility conduct an assessment to identify potential gaps when a patient is diagnosed with CDI?	91	7	2
**CDI Prevention Strategy 5: Engage the facility antibiotic stewardship program**	**Gap**	**Neutral**	**Strength**
For patients with new or recent CDI diagnosis, does your facility routinely review appropriateness of antibiotics prescribed for treatment of other conditions (eg, UTI, acute respiratory infections)?	67	26	7
Does your facility educate providers about the risk of CDI with antibiotics?	78	17	4
Does your facility educate patients/family members about the risk of CDI with antibiotics?	72	26	2
Does your facility routinely provide feedback to personnel on antibiotic use data (eg, appropriate agent, dose, duration, indication)?	83	15	2
Does your facility monitor the use of the following antibiotics that are high-risk for CDI:			
Fluoroquinolones (eg, ciprofloxacin, levofloxacin, ofloxacin, moxifloxacin)	93	4	2
Third- and Fourth-generation cephalosporins (eg, ceftazidime, cefepime)	93	4	2
Clindamycin	96	2	2
Does your facility use strategies to reduce the unnecessary use of the following antibiotics that are high risk for CDI:			
Fluoroquinolones (eg, ciprofloxacin, levofloxacin, ofloxacin, moxifloxacin)	98	0	2
Third- and Fourth-generation cephalosporins (eg, ceftazidime, cefepime)	98	0	2
Clindamycin	98	0	2

Note. CDI, *Clostridioides difficile* infection; UTI, urinary tract infection.

a n=46 hospitals that completed CDI TAP facility assessments.

b Gaps defined as ≥33% unknown, ≥50% unknown + no, or ≥50% unknown + never + rarely + sometimes.

c Strengths defined as ≥75% yes, or ≥75% always + often.

**Table 4.** Environmental Services (EVS) Assessment Questions for which at Least 50% of Hospitals Identified a Gap^a^

Question	% of Hospitals		
**Domain II. Environmental cleaning supplies**	**Gap** ^b^	**Neutral**	**Strength** ^c^
For non–English-speaking staff, are trainings given in the staff’s primary language?	85	0	15
Do EVS staff have input into the selection of cleaning and disinfection products?	60	30	10
**Domain III. Environmental cleaning practices**	**Gap**	**Neutral**	**Strength**
Are rooms with patients on isolation cleaned last on each unit?	75	5	20
**Domain IV. Contact Precautions/Hand hygiene**	**Gap**	**Neutral**	**Strength**
Do families and visitors adhere to use of gowns and gloves for patients on Contact Precautions?	75	25	0
Do families and visitors adhere to hand hygiene policies?	75	20	5

Note. EVS, environmental services.

a n=20 hospitals that completed EVS assessments.

b Gaps defined as ≥33% unknown, ≥50% unknown + no, or ≥50% unknown + never + rarely + sometimes.

c Strengths defined as ≥75% yes, or ≥75% always + often.

Common gaps identified from the assessments were related to CDI testing practices (strategy 2). This was demonstrated when 87% of the hospitals that collected assessments identified a gap when respondents were asked if providers avoid ordering *C. difficile* tests when the patient has a known cause for diarrhea (eg, history of laxatives). In addition, 98% identified a gap when respondents were asked if providers avoid testing for cure of CDI, and 70% identified a gap related to documenting an indication for *C. difficile* tests (Table 3).

Questions relating to infrastructure to support CDI prevention (strategy 4) were also among the most common gaps identified, as 91% of hospitals identified a gap regarding having a nurse champion for CDI prevention and 98% identified a gap regarding a physician champion. In addition, 91% of hospitals identified a gap regarding whether they conduct an assessment to identify potential prevention gaps when a patient is diagnosed with CDI (Table 3).

Most hospitals (93%–98%) also identified gaps related to monitoring or reducing the use of antibiotics that are high risk for CDI (Table 3). However, only ∼6% of respondents were responsible for prescribing medications. Nevertheless, 78% of hospitals identified a gap when asked if their facility educates providers about the risk of CDI with antibiotics, and 72% identified a gap related to educating patients and family members about the risk of CDI with antibiotics.

### Outcomes

After reviewing their Feedback Reports provided by the CDC, 15 hospitals (33%) submitted Gap Prioritization Worksheets and received customized hospital profiles. In total, 153 individual gaps were prioritized, and most of these 15 hospitals prioritized at least 1 gap within each of the 5 CDI prevention strategies (Table 2). The remaining 61 hospitals received hospital profiles containing similar feedback regarding the items most commonly identified as gaps across participating hospitals.

During the final meeting of the collaborative, 28 hospitals provided examples of how they “made a C-DIFFerence” during the collaborative (ie, preventive actions implemented). Among them, 15 hospitals (54%) reported developing infrastructure to support CDI prevention (eg, engaging key members in CDI task forces, establishing unit champions, case reviews of individual CDI episodes) and half reported interventions related to testing (eg, 2-step testing, testing algorithms, electronic alerts for patients on stool softeners) (Table 2).

During post-collaborative calls with 14 hospitals, primary factors contributing to their active participation and consistent engagement included keeping CDI reduction a priority (43%), leadership support (36%), and having a person dedicated to collaborative activities (36%). As a result of participation in the collaborative, 12 (86%) of the 14 follow-up hospitals reported implementing or planning to implement interventions related to testing, and interventions at 9 (64%) of these hospitals related to infrastructure to support CDI prevention (Table 2). These hospitals reported a total of 83 interventions implemented or planned for implementation. More than one-third of these interventions related to infrastructure to support CDI prevention (strategy 4; eg, establishing champions, review of individual CDI episodes); 22% related to CDI testing (strategy 2; eg, decision trees, 2-step testing); 16% related to environmental cleaning (strategy 3; eg, understanding of items cleaned by EVS and unit-level personnel); 13% related to antibiotic stewardship (strategy 5; eg, nurse engagement in antibiotic stewardship efforts); and 11% related to isolation and Contact Precautions (strategy 1; eg, daily baths with soap and water for patients with *C. diff*, patient hand hygiene). Furthermore, when asked what percentage of improvements in CDI prevention efforts at their hospital resulted from participation in the collaborative, the follow-up hospitals reported 53.1% on average (range, 30%–100%).

**Fig. 1.**
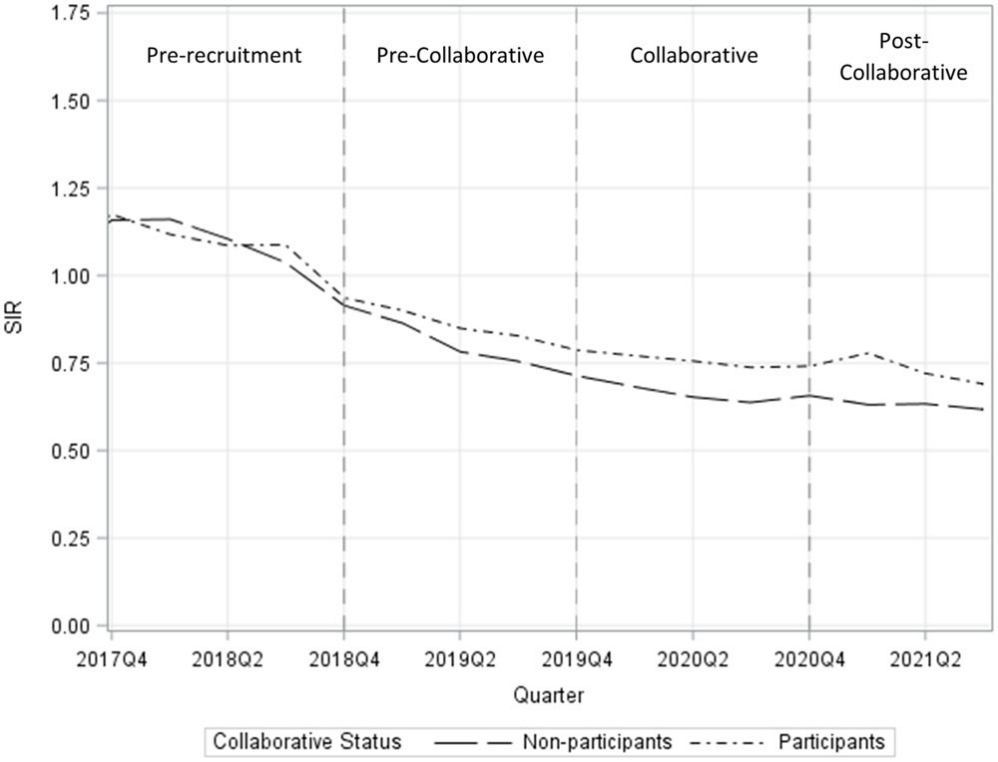
Standardized infection ratios (SIRs) by quarter for Collaborative participant hospitals (n = 76) and hospitals that met the recruitment criteria but did not participate (nonparticipants, n = 306).

Among all hospitals with a pre-collaborative SIR >0.70, 46 participating hospitals (61%) reported an SIR less than or equal to the Health and Human Services Healthy People 2030 SIR goal of 0.70 for the post-collaborative period.^7^ This percentage, however, was not significantly different from nonparticipants (64%; *P* = .56) or from the 15 hospitals that completed the TAP Strategy (47%; *P* = .29). Figure 1 displays the SIRs by quarter over time for participants (n = 76) and nonparticipants (n = 306). The SIR reductions across the groups were similar, but SIRs among participants were consistently higher. This finding aligns with the recruitment strategy of identifying hospitals with the greatest room for improvement. The SIR decrease between the pre-collaborative and post-collaborative periods was significant among participants (16.7%; 95% CI, 12.8%–20.4%; *P* < .001) but was less than that among nonparticipants (25.1%; 95% CI, 23.2%–27.0%; *P* < .001).

## Discussion

The Let’s Make a C-DIFFerence CDI prevention collaborative used data for action to identify hospitals for recruitment based on their CAD, or excess number of infections. Although infections at all hospitals remain important, this methodology allows public health partners to target resources to places where interventions may have the greatest impact.^6^ Through a series of monthly webinars and collaborative activities, participants were assisted in implementing the TAP Strategy to identify infection prevention gaps.

CDI testing practices were identified as a consistent theme throughout, and most hospitals identified potential gaps related to appropriate testing practices from the TAP facility assessments (eg, documentation of indication for tests, ordering *C. difficile* tests for patients with a known cause of diarrhea, testing for cure of CDI). Interventions used during and after the collaborative were often related to CDI testing practices, including implementing two-step testing, improving specimen stewardship (eg, laboratory rejection protocols, diagnostic decision trees), and creating electronic alerts. This finding highlights the complexities of CDI testing as well as the opportunity to provide additional support and directed guidance regarding testing, such as optimal test types and methodologies. Furthermore, the results of this collaborative influenced the decision to update the CDC CDI prevention strategies to include the consideration of 2-step testing to improve diagnostic accuracy when appropriateness of testing is an issue.^3^

Nurse and physician champions for CDI prevention were also among the most common gaps identified and prioritized among hospitals. This finding is consistent with data from TAP facility assessments for CAUTI and CLABSI, which highlighted that lack of awareness of champions may signal that champions do not exist within their hospitals or may not be facilitating prevention efforts effectively enough for staff to be aware of their roles.^8^ Having unit-level champions focusing on specific HAI prevention initiatives can help improve practices,^9–12^ and identification of champions for promotion of patient safety culture is an element of the World Health Organization (WHO) core components for effective infection prevention and control programs.^13^ To address this gap, the collaborative provided strategies for establishing infection prevention champions, outlined in four primary steps (ie, identify, train, empower, and support) on the CDC Infection Prevention Champions webpage.^14^ Although the implementation of infection prevention champions is not directly outlined in the CDI prevention strategies, champions should actively participate in multidisciplinary workgroups focusing on HAIs,^15^ which is an element of strategy 4 (develop infrastructure to support CDI prevention).

Although items related to antibiotic stewardship were least commonly prioritized and addressed by hospitals during the collaborative, assessment questions regarding antibiotic stewardship were among the largest gaps for most hospitals. However, this gap may be attributed to the lack of prescribers among respondents. Many hospitals described comprehensive antibiotic stewardship programs (ASPs), but this gap highlights the potential lack of awareness and involvement in stewardship efforts among nursing personnel. Nurse-based actions outlined in CDC Core Elements of Hospital ASPs include optimizing microbiology cultures, intravenous to oral medication transitions, and prompting discussions of antibiotic treatment, indication, and duration.^16–18^ Nurses also play an important role in patient education about appropriate antibiotic use^16,17^; however, >31% of nurses indicated “unknown” when asked whether the hospital educated patients and family members about the risk of CDI with antibiotics, and 20% responded “never”, “rarely”, or “sometimes”. Despite the critical role nurses play in patient care, perceived engagement of nurses in ASPs is low.^19,20^ As of 2019, 89% of hospitals reporting to the NHSN implemented all 7 core stewardship elements.^21^ Although this is encouraging, future efforts should focus on increasing engagement among nurses because they are optimally positioned throughout healthcare to guide appropriate antibiotic administration and improve patient care.

Upon review, SIRs were similar for the participant and nonparticipant hospitals in the pre-recruitment period, from which the CDI data were accessed to identify hospitals for recruitment. Recruitment took place mid-2019, which coincided with greater SIR improvements among non-participating hospitals, indicating that they may have chosen not to participate due to recent reduction progress being made. Although SIRs decreased from the pre-collaborative to post-collaborative periods among participants, this decrease was less than that among nonparticipants, and consistent with potential reductions nationwide. Although these preliminary data do not indicate that reductions were a direct result of the collaborative, they do highlight that the recruitment process was successful in enrolling those in greatest need (ie, with SIRs consistently above those of nonparticipants). Furthermore, the data indicate that even among hospitals with the highest SIRs and CADs nationally, they continued to make reductions during a global pandemic that has disrupted healthcare throughout the collaborative and post-collaborative periods. Future analysis would help measure the impact over a longer period; many participants reported that the COVID-19 pandemic affected their participation. Follow-up calls with select hospitals also revealed that many had implemented interventions within the post-collaborative period and planned to implement more in the future. Although 61% of participating hospitals collected >5,600 CDI TAP facility assessments to identify infection prevention gaps, most (78%) of these hospitals collected assessments prior to the start of the pandemic, and only 20% of participating hospitals reported reviewing their results and outlining potential interventions to address the gaps during the collaborative. Thus, while the hope is that hospitals will utilize collaborative resources and assessment data moving forward, the primary activities during the collaborative were focused on identifying gaps, not necessarily on implementing interventions across facilities.

This collaborative and summary are subject to several limitations. Participating hospitals did so voluntarily and represent a convenience sample among those identified for recruitment. Collaborative engagement varied, and many hospitals reported not being able to accomplish as much as they had hoped due to the COVID-19 pandemic. In addition, approximately half of the period used for analysis occurred during the COVID-19 pandemic, which affected routine practices, patient populations, staffing, and more, and potentially influenced the CDI data comparison between the pre-collaborative and post-collaborative periods. TAP facility assessments represent self-reported perceptions among hospital staff and items identified may not indicate true gaps in policies or practices. Furthermore, summary results including barriers, prioritized gaps, and interventions implemented were self-reported and voluntarily provided, and they were not obtained from all participants. NHSN data were incomplete for some hospitals due to optional reporting during the COVID-19 pandemic.

This summary demonstrates implementation of the comprehensive CDI Prevention Collaborative in hospitals targeted based on their CDI data. The consistently reported gaps including CDI testing practices, nurse and physician champions, and nurse engagement in ASPs highlight important areas in effective CDI prevention programs. Although preliminary analysis of post-collaborative data does not indicate greater reductions among participating hospitals, it does highlight that reductions across all groups continued to be made during a global pandemic when increases in many other HAIs have been reported.^2^ This summary also outlines potential future areas of focus for CDI prevention efforts based on >6,000 frontline assessments and reported challenges and barriers to prevention of one of the most common HAIs affecting hospitals and patients across the country.

## Supplementary material

For supplementary material accompanying this paper visit https://doi.org/10.1017/ash.2022.54. click here to view supplementary material
