# Supplementary material for: Making a C-DIFFerence: Implementation of a prevention collaborative to reduce hospital-onset Clostridioides difficile infection rates
Source: Antimicrob Steward Healthc Epidemiol. 2022 May 26;2(1):e87. doi: 10.1017/ash.2022.54 (PMC9726520; doi:10.1017/ash.2022.54)
Supplement: Supplementary file 1 [file S2732494X22000547sup001.pdf]

Supplemental: Targeted Assessment for Prevention (TAP) Strategy Overview and Summary of Collaborative Participation

TAP

The TAP Strategy is a framework for quality improvement that uses data for action to prevent HAIs by *targeting* locations with an excess burden of infections, *assessing* for infection prevention gaps, and implementing *prevention* strategies. The TAP Strategy is available for CAUTI, CLABSI, and CDI. All TAP Strategy tools and resources are publicly available for use at no cost. Optional technical assistance is also available from CDC upon request.

Target

- Generate TAP Reports using the National Healthcare Safety Network (NHSN)
- Identify facilities/units with excess HAIs using the Cumulative Attributable Difference (CAD) metric
- Review contextual factors and other data sources to target specific units

Resources

- NHSN TAP Reports
- TAP Dashboard
- TAP Report Instructional Video
- TAP Learning Series Video 1: Introduction to TAP
- TAP 'How To' Guides

Assess

- Assess targeted units for potential gaps in infection control using the TAP Facility Assessments
- Analyze responses and review results across units and groups to identify gaps
- Summarize potential gaps using TAP Feedback Reports

Resources

- TAP Facility Assessment Deployment Packet
- TAP Facility Assessment Tools
- TAP Learning Series Video 2: Deploying Assessments
- TAP Learning Series Video 3: Assessment Data

Prevent

- Review and prioritize gaps using the TAP Strategy Gap Prioritization Worksheet
- Access resources to aid in addressing identified gaps in the targeted locations to reduce infections
- Collaborate to implement prevention strategies, overcome barriers, and share successes

Resources

- TAP Strategy Gap Prioritization Worksheet
- TAP Learning Series Video 4: Prioritizing Gaps
- TAP Implementation Guides for CAUTI, CLABSI, and CDI
- TAP Prevention Prioritization Toolkit

Summary of Collaborative Participation

Target

- 400 hospitals identified for recruitment based on NHSN data → 382 hospitals contacted for recruitment → 76 hospitals enrolled for Collaborative participation
- 18 excluded due to active involvement in other CDC CDI prevention projects

Assess

- 46 hospitals deployed TAP CDI Facility Assessments, totaling 5,673 CDI assessments
- 20 hospitals deployed EVS Assessments, totaling 581 EVS assessments

Prevent

- 15 hospitals completed Gap Prioritization Worksheets, totaling 153 individual gaps prioritized → 14 hospitals participated in post-Collaborative calls → 83 reported interventions implemented or planned for implementation as a result of the Collaborative
